# Supplementary material for: Identification of Novel Hub Genes Associated with Psoriasis Using Integrated Bioinformatics Analysis
Source: Int J Mol Sci. 2022 Dec 4;23(23):15286. doi: 10.3390/ijms232315286 (PMC9737295; doi:10.3390/ijms232315286)
Supplement: Supplementary file 1 [file ijms-23-15286-s001.zip › ijms-1926146-supplementary.pdf]

Supplementary Table S1. GO and KEGG enrichment analysis of dataset GSE166388.

-	ID	Description	GeneRatio	BgRatio	*p* value	*p*.adjust	*q* value
BP	GO:0048562	embryonic organ morphogenesis	47/1017	288/18670	1.30e-11	3.90e-08	3.55e-08
BP	GO:0048568	embryonic organ development	60/1017	428/18670	1.49e-11	3.90e-08	3.55e-08
BP	GO:0048705	skeletal system morphogenesis	41/1017	239/18670	5.42e-11	9.47e-08	8.62e-08
CC	GO:0062023	collagen-containing extracellular matrix	57/1068	406/19717	3.81e-11	2.22e-08	2.04e-08
CC	GO:0000793	condensed chromosome	34/1068	223/19717	4.48e-08	1.30e-05	1.20e-05
CC	GO:0098687	chromosomal region	43/1068	349/19717	4.24e-07	8.23e-05	7.57e-05
MF	GO:0005201	extracellular matrix structural constituent	26/999	163/17697	1.49e-06	0.001	0.001
MF	GO:0003678	DNA helicase activity	16/999	81/17697	9.87e-06	0.004	0.004
MF	GO:0003697	single-stranded DNA binding	19/999	113/17697	1.74e-05	0.005	0.005
KEGG	hsa04270	Vascular smooth muscle contraction	20/426	135/8076	2.41e-05	0.007	0.006
KEGG	hsa04310	Wnt signaling pathway	19/426	160/8076	7.29e-04	0.073	0.067
KEGG	hsa04510	Focal adhesion	22/426	201/8076	8.79e-04	0.073	0.067

Supplementary Table S2. Data enrichment analysis using GO and KEGG GSE50790.

ONTOLOGY	ID	Description	GeneRatio	BgRatio	pvalue	*p*.adjust	qvalue
BP	GO:0070125	mitochondrial translational elongation	27/711	88/18670	1.10e-17	3.64e-14	3.43e-14
BP	GO:0070126	mitochondrial translational termination	27/711	89/18670	1.53e-17	3.64e-14	3.43e-14
BP	GO:0006415	translational termination	27/711	104/18670	1.20e-15	1.91e-12	1.80e-12
CC	GO:0000313	organellar ribosome	24/733	87/19717	6.29e-15	1.79e-12	1.67e-12
CC	GO:0005761	mitochondrial ribosome	24/733	87/19717	6.29e-15	1.79e-12	1.67e-12
CC	GO:0098798	mitochondrial protein complex	38/733	262/19717	6.28e-13	1.19e-10	1.11e-10
MF	GO:0003735	structural constituent of ribosome	27/689	202/17697	2.21e-08	1.78e-05	1.74e-05
MF	GO:0004298	threonine-type endopeptidase activity	6/689	21/17697	1.12e-04	0.030	0.029
MF	GO:0070003	threonine-type peptidase activity	6/689	21/17697	1.12e-04	0.030	0.029
KEGG	hsa03010	Ribosome	18/341	158/8076	1.13e-04	0.033	0.031
KEGG	hsa04932	Non-alcoholic fatty liver disease	16/341	150/8076	5.67e-04	0.083	0.077
KEGG	hsa05010	Alzheimer disease	29/341	369/8076	8.71e-04	0.085	0.079

Supplementary Table S3. GO and KEGG enrichment analysis of dataset GSE42632.

ONTOLOGY	ID	Description	GeneRatio	BgRatio	pvalue	*p*.adjust	qvalue
BP	GO:0042110	T cell activation	35/544	464/18670	3.06e-07	5.26e-04	4.58e-04
BP	GO:1903039	positive regulation of leukocyte cell-cell adhesion	22/544	218/18670	4.44e-07	5.26e-04	4.58e-04
BP	GO:0032496	response to lipopolysaccharide	28/544	330/18670	4.68e-07	5.26e-04	4.58e-04
CC	GO:0009897	external side of plasma membrane	29/566	393/19717	3.73e-06	0.002	0.002
CC	GO:0098978	glutamatergic synapse	26/566	349/19717	9.98e-06	0.002	0.002
CC	GO:0099240	intrinsic component of synaptic membrane	15/566	164/19717	7.96e-05	0.012	0.011
MF	GO:0005125	cytokine activity	25/541	220/17697	1.73e-08	1.19e-05	1.04e-05
MF	GO:0019955	cytokine binding	17/541	128/17697	3.69e-07	1.27e-04	1.11e-04
MF	GO:0008009	chemokine activity	10/541	49/17697	1.84e-06	3.76e-04	3.28e-04
KEGG	hsa04060	Cytokine-cytokine receptor interaction	37/283	295/8076	8.04e-12	2.28e-09	2.12e-09
KEGG	hsa04061	Viral protein interaction with cytokine and cytokine receptor	14/283	100/8076	8.98e-06	0.001	0.001

KEGG	hsa03320	PPAR signaling pathway	12/283	78/8076	1.49e-05	0.001	0.001

Supplementary Table S4. Information on 57 genes from the intersection of the three Datasets.

Numbers	GSE1|GSE2|GSE3	Full name	Location	Highest median expression	Ensembl gene ID
1	ALDH1A3	Aldehyde dehydrogenase 1 family member A3	hg38 chr15:100877714-100916626	63.65 TPM in Prostate	ENSG00000184254.16
2	AREG	Amphiregulin	hg38 chr4:74445134-74455009	53.38 TPM in Adipose-Visceral (Omentum)	ENSG00000109321.10
3	FAM43A	Family with sequence similarity 43 members A	hg38 chr3:194686544-194689037	95.51 TPM in Spleen	ENSG00000185112.5
4	HOTAIR	HOX transcript antisense	hg38 chr12:53962308-53974956	27.05 TPM in Artery—Tibial	ENSG00000228630.5
5	CMAHP	Cytidine monophospho-N-acetylneuraminic acid hydroxylase, pseudogene	hg38 chr6:25081320-25166555	37.23 TPM in Thyroid	ENSG00000168405.15
6	AQP9	Aquaporin 9	hg38 chr15:58138169-58185911	596.30 TPM in Whole Blood	ENSG00000103569.9
7	IL-1β	Interleukin 1 beta	hg38 chr2:112829751-112836903	24.53 TPM in Whole Blood	ENSG00000125538.11
8	ASPM	Assembly factor for spindle microtubules	hg38 chr1:197084128-197146694	25.05 TPM in Cells-EBV-transformed lymphocytes	ENSG00000066279.17
9	FAM111B	Family with sequence similarity 111 member B	hg38 chr11:59107185-59127410	51.31 TPM in Cells-EBV-transformed lymphocytes	ENSG00000189057.10
10	HMMR	Hyaluronan mediated motility receptor	hg38 chr5:163460203-163491945	56.76 TPM in Cells-EBV-transformed lymphocytes	ENSG00000072571.19
11	CEP55	Centrosomal protein 55	hg38 chr10:93496632-93529092	54.03 TPM in Cells-EBV-transformed lymphocytes	ENSG00000138180.15

12	ESCO2	Establishment of sister chromatid cohesion N-acetyltransferase 2	hg38 chr8:27771949-27812640	17.01 TPM in Cells-EBV-transformed lymphocytes	ENSG00000171320.14
13	CYP2J2	Cytochrome P450 family 2 subfamily J member 2	hg38 chr1:59893308-59926790	42.36 TPM in Heart-Atrial Appendage	ENSG00000134716.9
14	MCM10	Minichromosome maintenance 10 replication initiation factor	hg38 chr10:13161554-13211104	17.44 TPM in Cells-EBV-transformed lymphocytes	ENSG00000065328.16
15	PBK	PDZ binding kinase	hg38 chr8:27809620-27838095	81.37 TPM in Testis	ENSG00000168078.9
16	TOP2A	DNA topoisomerase II alpha	hg38 chr17:40388516-40417950	150.75 TPM in Cells-EBV-transformed lymphocytes	ENSG00000131747.14
17	LIPG	Lipase G, endothelial type (LIPG)	hg38 chr18:49560699-49599182	95.40 TPM in Thyroid	ENSG00000101670.11
18	TNIP3	TNFAIP3 interacting protein 3	hg38 chr4:121131408-121227466	1.43 TPM in Cells-EBV-transformed lymphocytes	ENSG00000050730.15
19	IFI44	Interferon induced protein 44	hg38 chr1:78649796-78664078	208.70 TPM in Cells-EBV-transformed lymphocytes	ENSG00000137965.10
20	CDKN3	Cyclin dependent kinase inhibitor 3	hg38 chr14:54396849-54420218	176.20 TPM in Testis	ENSG00000100526.19
21	HELZ2	Helicase with zinc finger 2	hg38 chr20:63558086-63574239	24.81 TPM in Cells-EBV-transformed lymphocytes	ENSG00000130589.16
22	PCLAF	PCNA clamp associated factor	hg38 chr15:64364311-64387687	29.71 TPM in Cells-EBV-transformed lymphocytes	ENSG00000166803.11
23	SERPINB3	Serpin family B member 3	hg38 chr18:63655197-63661963	574.00 TPM in Esophagus-Mucosa	ENSG00000057149.15
24	GDA	Guanine deaminase	hg38	36.99 TPM in	ENSG0000

			chr9:72114595-72257193	Brain-Frontal Cortex (BA9)	0119125.16
25	GPR68	G protein-coupled receptor 68	hg38 chr14:91232532-91253925	20.72 TPM in Pituitary	ENSG00000119714.10
26	PAPLN	Papilin, proteoglycan like sulfated glycoprotein	hg38 chr14:73237497-73274640	108.20 TPM in Nerve—Tibial	ENSG00000100767.15
27	KIF23	Kinesin family member 23	hg38 chr15:69415994-69448427	37.33 TPM in Cells-EBV-transformed lymphocytes	ENSG00000137807.13
28	DLGAP5	DLG associated protein 5	hg38 chr14:55148112-55191678	65.98 TPM in Cells-EBV-transformed lymphocytes	ENSG00000126787.12
29	SPC25	SPC25 component of NDC80 kinetochore complex	hg38 chr2:168834132-168913371	26.91 TPM in Cells-EBV-transformed lymphocyte	ENSG00000152253.8
30	LYPD6B	LY6/PLAUR domain containing 6B	hg38 chr2:149038107-149215262	50.51 TPM in Skin- Not Sun Exposed (Suprapubic)	ENSG00000150556.16
31	NEK2	NIMA related kinase 2	hg38 chr1:211658657-211675630	52.47 TPM in Testis	ENSG00000117650.12
32	MELK	Maternal embryonic leucine zipper kinase	hg38 chr9:36572862-36677683	46.03 TPM in Cells-EBV-transformed lymphocytes	ENSG00000165304.7
33	DEPDC1B	DEP domain containing 1B	hg38 chr5:60596912-60700190	26.82 TPM in Cells-EBV-transformed lymphocytes	ENSG00000035499.12
34	NETO2	Neuropilin and tolloid like 2	hg38 chr16:47077703-47143997	13.40 TPM in Cells-EBV-transformed lymphocytes	ENSG00000171208.9
35	UBE2C	Ubiquitin conjugating enzyme E2 C	hg38 chr20:45812576-45816957	144.45 TPM in Cells-EBV-transformed lymphocytes	ENSG00000175063.16

36	FAM13A-AS1	FAM13A antisense RNA 1	hg38 chr4:88709789-88730103	30.09 TPM in Brain-Cerebellum	ENSG00000248019.2
37	ATP8A1	ATPase phospholipid transporting 8A1	hg38 chr4:42408373-42657105	64.08 TPM in Thyroid	ENSG00000124406.16
38	SERPINB9	Serpin family B member 9	hg38 chr6:2887266-2903280	159.70 TPM in Cells-EBV-transformed lymphocytes	ENSG00000170542.5
39	BUB1	BUB1 mitotic checkpoint serine/threonine kinase	hg38 chr2:110637698-110678114	88.96 TPM in Cells-EBV-transformed lymphocytes	ENSG00000169679.14
40	RRM2		hg38 chr2:10122328-10131419	174.50 TPM in Cells-EBV-transformed lymphocytes	ENSG00000171848.13
41	DNASE1L3	Deoxyribonuclease 1 like 3	hg38 chr3:58192257-58214697	106.00 TPM in Spleen	ENSG00000163687.13
42	CDCA2	Cell division cycle associated 2	hg38 chr8:25458997-25507920	36.57 TPM in Testis	ENSG00000184661.13
43	ABCA11P	ATP binding cassette subfamily A member 11, pseudogene	hg38 chr4:425435-427882	9.73 TPM in Pituitary	ENSG00000251595.7
44	NDC80	NDC80 kinetochore complex component	hg38 chr18:2571511-2616635	36.81 TPM in Cells-EBV-transformed lymphocytes	ENSG00000080986.12
45	CCNB2	Cyclin B2	hg38 chr15:59105078-59125045	79.42 TPM in Testis	ENSG00000157456.7
46	TK1	Thymidine kinase 1	hg38 chr17:78174075-78187233	118.55 TPM in Cells-EBV-transformed lymphocytes	ENSG00000167900.11
47	UBE2T	Ubiquitin conjugating enzyme E2 T	hg38 chr1:202331657-202341980	92.91 TPM in Cells-EBV-transformed lymphocytes	ENSG00000077152.9
48	CYP3A5	cytochrome P450 family 3 subfamily A member 5	hg38 chr7:99648194-	155.90 TPM in Liver	ENSG00000106258.13

			99679998		
49	UPP1	Uridine phosphorylase 1	hg38 chr7:48088628-48108733	142.00 TPM in Whole Blood	ENSG00000183696.13
50	BIRC5	Baculoviral IAP repeat containing 5	hg38 chr17:78214186-78225636	58.58 TPM in Cells-EBV-transformed lymphocytes	ENSG00000089685.14
51	FOS	Fos proto-oncogene, AP-1 transcription factor subunit	hg38 chr14:75278774-75282230	1112.00 TPM in Fallopian Tube	ENSG00000170345.9
52	ISG20	Interferon stimulated exonuclease gene 20	hg38 chr15:88636153-88656483	98.66 TPM in Cells-EBV-transformed lymphocytes	ENSG00000172183.14
53	SLC7A5	Solute carrier family 7 member 5	hg38 chr16:87830023-87869488	150.70 TPM in Testis	ENSG00000103257.8
54	KIF20A	Kinesin family member 20A	hg38 chr5:138178998-138187647	39.53 TPM in Cells-EBV-transformed lymphocytes	ENSG00000112984.11
55	TTC39A	Tetratricopeptide repeat domain 39A	hg38 chr1:51287258-51345116	35.54 TPM in Testis	ENSG00000085831.15
56	CDCA3	Cell division cycle associated 3	hg38 chr12:6844793-6852066	29.41 TPM in Testis	ENSG00000111665.11
57	LAMP3	Lysosomal associated membrane protein 3	hg38 chr3:183122213-183163839	105.05 TPM in Cells-EBV-transformed lymphocytes	ENSG00000078081.7
